# Status of insecticide resistance in malaria vectors in Kwale County, Coastal Kenya

**DOI:** 10.1186/s12936-017-2156-6

**Published:** 2018-01-05

**Authors:** Caroline W. Kiuru, Florence Awino Oyieke, Wolfgang Richard Mukabana, Joseph Mwangangi, Luna Kamau, Damaris Muhia-Matoke

**Affiliations:** 1 School of Biological Sciences, University of Nairobi, P.O. Box 30197, Nairobi, 00100 Kenya; 2 Department of Biological Sciences, Pwani University, Kilifi, Kenya; 3 Man & Well-Being Research Office, Science for Health, P.O. Box 44970, Nairobi, 00100 Kenya; 4 KEMRI-Centre for Geographic Medicine Research, Kilifi, Kenya; 5 KEMRI-Centre for Biotechnology Research and Development, Nairobi, Kenya

**Keywords:** Malaria vectors, Insecticide resistance, *kdr*

## Abstract

**Background:**

The strategy for malaria vector control in the context of reducing malaria morbidity and mortality has been the scale-up of long-lasting insecticidal nets to universal coverage and indoor residual spraying. This has led to significant decline in malaria transmission. However, these vector control strategies rely on insecticides which are threatened by insecticide resistance. In this study the status of pyrethroid resistance in malaria vectors and it’s implication in malaria transmission at the Kenyan Coast was investigated.

**Results:**

Using World Health Organization diagnostic bioassay, levels of phenotypic resistance to permethrin and deltamethrin was determined. *Anopheles arabiensis* showed high resistance to pyrethroids while *Anopheles gambiae* sensu stricto (s.s.) and *Anopheles funestus* showed low resistance and susceptibility, respectively. *Anopheles gambiae* sensu lato (s.l.) mosquitoes were further genotyped for L1014S and L1014F kdr mutation by real time PCR. An allele frequency of 1.33% for L1014S with no L1014F was detected. To evaluate the implication of pyrethroid resistance on malaria transmission, *Plasmodium falciparum* infection rates in field collected adult mosquitoes was determined using enzyme linked immunosorbent assay and further, the behaviour of the vectors was assessed by comparing indoor and outdoor proportions of mosquitoes collected. Sporozoite infection rate was observed at 4.94 and 2.60% in *An. funestus* s.l. and *An. gambiae* s.l., respectively. A higher density of malaria vectors was collected outdoor and this also corresponded with high *Plasmodium* infection rates outdoor.

**Conclusions:**

This study showed phenotypic resistance to pyrethroids and low frequency of L1014S *kdr* mutation in *An. gambiae* s.l. The occurrence of phenotypic resistance with low levels of *kdr* frequencies highlights the need to investigate other mechanisms of resistance. Despite being susceptible to pyrethroids *An. funestus* s.l. could be driving malaria infections in the area.

## Background

In the last decade, there have been global efforts to reduce malaria morbidity and mortality through different programmes [1]. In Kenya, the main strategies employed by the National Malaria Control Programme have been: (1) scaling up of vector control interventions; (2) timely diagnosis and effective treatment using artemisinin-based combination therapy (ACT); and (3) intermittent preventive treatment for pregnant women (IPTP) [2]. For vector control, the World Health Organization (WHO) recommends long-lasting insecticidal nets (LLINs), indoor residual spraying (IRS) and larval source management [3, 4]. The most common and widespread of these methods is the use of IRS and LLINs, with LLINs being more dominant due to ease of distribution and low cost associated with their roll out [5].

Pyrethroids remain the only class of insecticides recommended for the treatment of LLINs. This arises from their low toxicity to humans and their mode of action which entails rapid and persistent effects against mosquitoes [6]. Until an alternative is obtained, vector control using LLINs remains heavily dependent on pyrethroids.

In Kenya, a mass bed net distribution campaign in malaria endemic areas was held in 2006, where LLINs given to pregnant women and children below the age of 5 years attained a coverage of 60% [7]. In 2012, a near universal coverage was attained with another mass distribution of LLINs, where the goal of one net in every two people in a household was reached [8]. This increased ITN coverage coupled to the other malaria control strategies has seen reduction in malaria transmission in many localities. Unfortunately, the sustainability of vector control using LLINs remains questionable due to insecticide resistance. Already the last decade has seen increased reports of resistance to pyrethroids in at least 27 countries in Africa [9]. In Kenya, resistance has been reported in Western Kenya, with no report on the current state of insecticide resistance in the Kenyan Coast despite intensified vector control in the area [10–12].

The main malaria vectors in the Kenyan Coast are; *Anopheles funestus* sensu lato (s.l.) and *Anopheles gambiae* s.l., both of which are complex species [13]. Members of both complexes exhibit variation in their biology making it difficult to have a universal control. With LLINs targeting malaria vectors biting indoors, the occurrence of resistance could greatly impact malaria transmission dynamics. Changes in species composition, as well as, changes in their role in transmission have been reported in relation to increased bed-net use and coverage [14, 15]. With the increased use of pyrethroids for LLINs, IRS and in agriculture mosquitoes are subjected to insecticide pressure thereby, increasing their probability of resistance [16].

In the light of the increased bed-net coverage and the likelihood of this serving as a potential selective pressure for malaria vectors, this study sort to assess the level of phenotypic and genotypic resistance in Kwale County in the Kenyan Coast. In an effort to understand the implication of resistance on malaria transmission, species composition, sporozoite infection and indoor/outdoor proportions of malaria vectors was assessed.

## Methods

### Study area

The study was conducted in South Coast Kenya in Kwale County. The study area has been previously described [17]. *Anopheles funestus* s.l. and *An. gambiae* s.l. are the main malaria vectors in the area [18, 19]. They occur all year round, with peak season during the rainy season [18]. In the area, 50% of households have universal ITN coverage (≤ 2 persons per ITN). Generally, for the Kenyan Coast an increase in malaria prevalence from 4 to 8% since 2010 has been reported. Sampling was done in two villages; Marigiza (Latitude − 4.443036, Longitude 39.461887) and Kidomaya (Latitude − 4.578639, Longitude 39.157574) which are about 50 km apart representing the Coastal plain and Coastal estuarine habitats, respectively. The history of bed net use in Kidomaya dates back to 1998 where all households were provided with insecticide-treated nets (ITNs) as part of a clinical trial [20]. After this, the two villages have received parallel distribution of LLINs through the National Malaria Control Programme by mass distribution campaigns held in 2006 and 2012.

### Mosquito collection and rearing

Mosquitoes were collected in July and August 2015, which corresponds to the dry season. Adult mosquitoes were collected using light traps, which have been reported to be efficient in collecting host-seeking mosquitoes [19]. Additionally, Light traps were supplemented with Prokopack aspirator (John W. Hock Co., Gainesville, FL, USA) to capture indoor resting mosquitoes.

In each trapping night, light traps were set up both inside and outside three randomly selected houses in each village between 1800 and 1700 h. Indoor traps were set up at the foot side of the bed 1 m off the ground and approximately 1.5 m from the place of sleep [21]. The outdoor traps were placed at least 5 m from houses containing the indoor light traps. The traps were removed the following morning between 0600 and 0700 h. To boost the number of blood-fed mosquitoes collected, aspiration was done in the same and nearby houses within 500 m radius in the morning between 0700 and 0900 h using Prokopack aspirator with a view of maximum sampling. Live mosquitoes collected by light traps and aspirators were transferred into paper cups, provided with 6% sucrose and stored in a cool box for transportation to Msambweni Hospital Research laboratory for further processing.

Adult mosquitoes collected were sorted according to their sex and physiological status i.e. as gravid, half gravid, blood fed and unfed. Live mosquitoes that were gravid, half gravid and blood-fed were kept in paper cups in the insectary. They were provided with 6% sucrose, when fully gravid they were transferred to individual egg laying tubes. The egg laying tubes were perforated 1.5 ml eppendorf tubes lined with a moistened strip of filter paper [22]. Eggs from individual females were reared in separate trays to obtain iso-female families.

Larvae collection was done in all identified water bodies in each village. Approximately five larval habitats were sampled in each village per week. Initially, larvae were collected using the standard dipping method whereby, ten dips were made per potential larval habitat using a standard 350 ml dipper [23]. As the densities of *Anopheles* larvae and the number of habitats were low, the number of dips was increased and larvae were collected exhaustively from the habitats. This adjustment was in accordance to WHO guidelines for monitoring insecticide resistance, that larval collections be made from a number of different breeding habitats to avoid collecting larvae from single egg batches [24]. Collected larvae were transported in Whirl–Pak^®^ bags to the laboratory where larvae from the same village were pooled together and sorted by their instar stages. Larvae were maintained using Tetramin^®^ baby fish food.

### Insecticide susceptibility bioassays

Female, F0 adults reared from larval collections were used for the bioassay. This is because no F1 adults were obtained from field collected adults due to high larval mortalities. Non-blood fed 3–5 days old female adults were exposed to 0.75% permethrin and 0.05% deltamethrin or control papers impregnated with silicone oil at temperatures of 25 ± 2 °C and 70–80% relative humidity according to WHO insecticide susceptibility test guidelines [24]. *Anopheles* mosquitoes in batches of 18–25 were placed in holding tubes for 1 h after which any moribund mosquito was removed before being transferred to exposure tubes lined with insecticide or silicone oil impregnated papers. The tubes were held in vertical position and the knockdown rate recorded at intervals of 10, 15, 20, 30, 40, 50 and 60 min. After 60 min the mosquitoes were transferred to holding tubes, maintained on 6% glucose and mortality rate determined 24 h post exposure. Laboratory reared *An. gambiae* sensu stricto (s.s.) Kisumu strain were exposed to each insecticide as positive control while 20–25 field collected adults were exposed to control papers as negative control and used in correcting mortality rate using the Abbotts’s formula [24]. Mortality rate was calculated by expressing the total number of dead mosquitoes from all four replicates for an individual insecticide as a percentage of the total exposed.

### Mosquito identification and sibling differentiation

Filed collected adults and adults emerging from field collected larvae were identified morphologically to species level [25] and preserved on silica gel granules at room temperature. The field collected adults were later dissected into three portions; (1) head and thorax, (2) legs and wings, and (3) abdomen. For field collected adults, genomic DNA was extracted from the legs and wings. The other body sections were used for other analysis such as sporozoite ELISA analysis. For adults emerging from field collected larvae, genomic DNA was extracted from the whole body, and subjected to species ID and KDR analysis [26]. The DNA was used for sub-species identification for *An. gambiae* [27] and *An. funestus* [28] complexes by conventional polymerase chain reaction (PCR). For the *An. gambiae* complex, further analysis to differentiate M and S molecular forms was not performed, as only the S form has been reported to be present in East Africa [29].

### Detection of *kdr* mutations

For the *An. gambiae* complex, the genomic DNA was further used to test for the presence of point mutations at the position 1014 of the voltage gated sodium channel by real time PCR, TaqMan probe based assay [30]. Both the leucine to phenylanine substitution (*kdr* west allele) and leucine to serine substitution (*kdr* east allele) were tested.

### Sporozoite analysis

The heads and thoraces of individual anopheline females were tested for the presence of *Plasmodium falciparum* circumsporozoite antigen using sandwich enzyme-linked immunosorbent assay (ELISA) [31]. The infection rate was calculated as the proportion of infected mosquitoes.

### Data analysis

Data was entered in Microsoft Excel 2010 and analysed using R software, version 3.3.2. Resistance was determined using the WHO classification of mortality rate where 98–100% mortality indicates susceptibility, 90–97% suggests possible resistance for which further investigation is required while < 90% is considered resistance. Frequency counts for categorical data were compared using Pearsons’s Chi square test performed at 0.05 level of significance.

## Results

### Species composition

A total of 1101 *Anopheles* adults and larvae were collected from the two villages, Marigiza (520) and Kiodomaya (581). Of these, 63.03% (n = 694) were collected as adults while 36.97% (n = 407) were collected as larvae. From the adults collected, 154 were gravid, half-gravid or blood-fed, and were placed in oviposition tubes. The oviposited eggs hatched but did not survive past the 2nd larval instar. It is worth noting that only two blood-fed *An. gambiae* s.l. were collected and only one laid eggs which hatched but also did not survive.

Overall, the proportion of *An. funestus* s.l. collected was higher (64.40%) compared to *An. gambiae* s.l. (33.97%) and other secondary malaria vectors (1.63%) (χ^2^ = 650.72, df = 2, p < 0.001). The secondary malaria vectors collected include; *Anopheles squamosus* (n = 7), *Anopheles coustani* (n = 5), *Anopheles pharoensis* (n = 5) and *Anopheles pretoriensis* (n = 1) (Table 1). The proportion of *An. gambiae* s.l. collected in Kidomaya was significantly higher compared to Marigiza (χ^2^ = 13.10, df = 1, p = 0.003) while no differences in *An. funestus* s.l. was observed between the two villages. Amongst the 374 *An. gambiae* s.l. collected, 88.24% were *Anopheles arabiensis*, 4.81% were *An. gambiae* s.s. while 6.95% did not amplify (Table 1).

**Table 1:** Species composition of *Anopheles* mosquitoes collected in Marigiza and Kidomaya villages in Kwale County, Coastal Kenya

Species	Sibling species ID	Kidomaya	Marigiza	Total
*An. gambiae*	*An. arabiensis*	183	147	330
	*An. gambiae* s.s.	16	2	18
	Not amplified	23	3	26
	Total	222	152	374
*An. funestus*	*An. funestus* s.s.	250	289	539
	*An. leesoni*	24	1	25
	*An. parensis*	13	8	21
	*An. rivulorum*	2	9	11
	*An. vaneedeni*	1	4	5
	Hybrids	2	4	6
	Not amplified	63	39	102
	Total	355	354	709
*An. coustani*		3	2	5
*An. pharoensis*		0	5	5
*An. squamosus*		0	7	7
*An. pretoriensis*		1	0	1

Out of 709 *An. funestus* s.l. collected, PCR species identification revealed that 76.02% were *An. funestus* s.s., 3.53% *Anopheles leesoni*, 2.96% *Anopheles parensis*, 1.55% *Anopheles rivulorum*, 0.71% *Anopheles vaneedeni* and 0.85% hybrids, while 14.39% did not amplify. Hybrids were identified based on production of two bands corresponding to two different sibling species after PCR amplification. The composition of the hybrids was: one *An. parensis*/*An. leesoni*, one *An. funestus*/*An. parensis* and four *An. vaneedeni*/*An. parensis*. However, the hybrids identified in this study will need to be analysed further as the occurrence of hybrids has been associated with sequence similarity between other species and members of *An. funestus* complex in the internal transcribed spacer region 2 of the rDNA. The dominant *An. funestus* sibling species was *An. funestus* s.s. in both villages.

### Outdoor and indoor collections

Overall, no difference was observed between the total numbers of mosquitoes collected outdoor and indoor. In the interest of fairly comparing indoor and outdoor proportions we considered only mosquitoes collected by light traps for analysis in this section as aspirators were not used for outdoor collections. A higher number of mosquitoes was collected outdoor (76.13%, n = 370) compared to indoor (23.78%, n = 116) (χ^2^ = 132.75, df = 1, p < 0.001). Only *An. rivulorum* had higher proportions indoor compared to outdoor (Table 2).

**Table 2:** Total number and proportion of mosquitoes collected by light trap indoors and outdoors

Species	Sibling species	Total	Indoor proportion (%)	Outdoor proportion (%)
*An. funestus* s.l.	–	397	24.69	75.31
	*An. funestus* s.s.	280	22.14	77.86
	*An. leesoni*	24	16.67	83.33
	*An. parensis*	14	28.57	71.43
	*An. rivulorum*	5	60	40
	*An. vaneedeni*	3	66.67	33.33
	Hybrids	3	0	100
	Not amplified	68	33.82	66.18
*An. gambiae* s.l.	–	74	22.97	77.03
	*An. arabiensis*	55	23.64	76.36
	*An. gambiae* s.s.	2	50	50
	Not amplified	17	17.65	82.35
*An. coustani*	–	5	0	100
*An. pharoensis*	–	5	0	100
*An. squamosus*	–	4	25	75
*An. pretoriensis*	–	1	0	100

### Insecticide susceptibility bioassay

A total of 407 F0 female adult mosquitoes aged 3–5 days old raised from larvae were used to test for susceptibility to deltamethrin and permethrin. From the 407, 72.24% (n = 294) were *An. gambiae* s.l., 27.03% (n = 110) *An. funestus* s.l. and 0.74% (n = 3) *An. squamosus*. Of these, 155 and 201 were exposed to deltamethrin and permethrin impregnated papers, respectively, while 51 were exposed to the control papers impregnated with silicone oil. From the 356 exposed to insecticide treated papers, an overall mortality of 76.97% was observed with a mortality rate of 75.48 and 78.11% for deltamethrin and permethrin, respectively. The mortality rate for *An.* gambiae s.s. Kisumu stain was 100% indicating full susceptibility to the insecticides and therefore confirming the effectiveness of the insecticide impregnated papers. No mortality was observed for the negative control with silicone oil impregnated papers, thus there was no need to correct for natural causes of mortality using the Abbott’s formula.

All *An*. *funestus* s.l. tested showed 100% susceptibility to both deltamethrin (n = 54) and permethrin (n = 41) (Table 3). For *An. gambiae* s.l. overall mortality upon exposure to pyrethroids was 68.58% with mortality to deltamethrin and permethrin being 62.38% (n = 101) and 72.50% (n = 160), respectively both of which indicate resistance to pyrethroids. For the specific *An. gambiae* s.l. species, *An. arabiensis* exhibited an overall mortality to pyrethroids of 66.52% with a higher mortality to permethrin (69.93%) compared to deltamethrin (61.05%). Compared to *An. arabiensis*, *An. gambiae* s.s. showed a higher overall mortality to pyrethroids 92.86 with 100% mortality to permethrin and 75% mortality to deltamethrin. This indicates that *An. gambiae* s.s. are susceptible to permethrin and resistant to deltamethrin. As no differences in mortality rate was observed between the two villages, Kidomaya and Marigiza mortality rate data was analysed together for both villages (Table 3).

**Table 3:** Mortality rate in female *Anopheles* mosquitoes exposed to deltamethrin and permethrin

Insecticide	Species	Sibling species	Mortality
Deltamethrin	*An. funestus* s.l.	–	54 (100)
		*An. funestus* s.s.	49 (100)
		*An. vaneendeni*	1 (100)
		Not amplified	4 (100)
	*An. gambiae* s.l.	–	101 (62.38)
		*An. arabiensis*	95 (61.05)
		*An. gambiae* s.s.	4 (75)
		Not amplified	2 (100)
Permethrin	*An. funestus* s.l.	–	41 (100)
		*An. funestus* s.s.	38 (100)
		Not amplified	3 (100)
	*An. gambiae* s.l.	–	160 (72.50)
		*An. arabiensis*	143 (69.93)
		*An. gambiae* s.s.	12 (100)
		Not amplified	5 (80)

Number outside parenthesis is the total number exposed to insecticide impregnated papers. Number inside parenthesis is the mortality rate in %

### Knockdown resistance mutations

Three hundred *An.* gambiae s.l. were genotyped for *kdr*-East (L1014S) and *kdr*-West (L1014F) mutations. Out of the 300 mosquitoes, 53 were from field collected adults while 247 were from the adults used for the bioassay, 79 of which had exhibited the resistance phenotype upon exposure to pyrethroid impregnated papers. Only L1014S mutation was detected in five *An*. *gambiae* s.s. 3 of which were homozygous while 2 were heterozygous for the L1014S allele. Of the 79 mosquitoes that had exhibited the resistance phenotype after exposure to pyrethroids only one *An. gambiae* s.s. showed genotypic resistance and was heterozygous for the L1014S allele (Table 4).

**Table 4:** Frequency of Knockdown resistance allele in relation to phenotypes determined by WHO susceptibility bioassay in *Anopheles gambiae* s.s.

Bioassay phenotype	n	L1014S kdr genotype			F (kdr)
		RR	RS	SS	
Resistant	78	0	1	77	0.0064
Susceptible	169	3	1	165	0.0207

R represents the resistant allele, S represents the wild type/susceptible allele, n is the total number tested and F is the frequency of the *kdr* allele. The resistant bioassay phenotype refers to mosquitoes that were alive 24 h post-exposure to either deltamethrin or permethrin while susceptible phenotype refers to those that were dead

### Sporozoite infection rates

Six hundred and fifty-nine mosquitoes were tested for the presence of *P. falciparum* parasites. Thirty tested positive giving an overall infection rate of 4.55%. The infection rate was higher in *An. funestus* 4.94% (n = 567) compared to *An. gambiae* 2.60% (n = 77), these did not differ significantly (χ^2^ = 0.8364, p = 0.36). For secondary malaria vectors collected no sporozoite infection was detected. There was no difference observed between outdoor infection rate (4.35%, n = 16) and indoor infection rate (4.81%, n = 14). From the indoor collected mosquitoes, only *An. funestus* s.l. (5.20%, n = 14) were infected while for outdoor collected mosquitoes both *An. funestus* s.l. (4.70%, n = 1) and *An. gambiae* s.l. (3.57%, n = 2) were infected. The infection rate of the specific sibling species is shown in Table 5.

**Table 5:** *Plasmodium falciparum* sporozoite infection rate of *Anopheles* mosquitoes from Kwale, Coastal Kenya

Species	Sibling species	Total tested	% positive
*An. funestus* s.l.	–	567	4.94
	*An. funestus* s.s.	413	4.84
	*An. leesoni*	25	4.00
	*An. parensis*	20	5.00
	*An. rivulorum*	10	0
	*An. vaneedeni*	4	0
	Hybrids	6	33.33
	Not amplified	89	3.74
*An. gambiae* s.l.	–	75	2.60
	*An. arabiensis*	55	3.51
	*An. gambiae* s.s.	2	0
	Not amplified	18	0
*An. coustani*	–	5	0
*An. pharoensis*	–	5	0
*An. squamosus*	–	4	0
*An. pretoriensis*	–	1	0

## Discussion

The present study documents a mortality rate of 75.48 and 78.11% for deltamethrin and permethrin, respectively in malaria vectors in Kwale County. For the assessment of phenotypic resistance, WHO classifies a population into three categories based on their percentage mortality or susceptibility: A population with 100–98% mortality is regarded susceptible, 97–90% mortality indicates possible resistance that needs confirmation either using more bioassays or assessing the level of resistant genes while < 90% indicates resistance [24]. Based on this classification, this study reveals presence of phenotypic resistance to pyrethroids in *An. arabiensis*, possible resistance in *An. gambiae* s.s. and susceptibility in *An. funestus* s.l. In comparison to an earlier study in the Kenyan Coast [32] that showed low level of resistance (83–93%) to deltamethrin in *An. gambiae* s.l., this study shows high levels of resistance (62.38%). This increased resistance levels might be as a result of selection pressure due to increased ITN coverage. However, the contribution of agricultural insecticides should not be ignored.

Though high levels of phenotypic resistance were exhibited, the levels of *kdr* allele frequency were very low (1.33%). Surprisingly, while *An. arabiensis* was more resistant to deltamethrin and permethrin compared to *An. gambiae* s.s. no *kdr* mutation was detected in *An*. *arabiensis* suggesting that other mechanisms could be contributing to the resistance phenotype observed.

For the species composition of malaria vectors this study shows higher densities of *An. funestus* s.l. in comparison to *An. gambiae* s.l. This is in line with previous studies that have reported changes in species composition with relative increase in *An. funestus* s.l. compared to *An. gambiae* s.l. These changes have been alluded to insecticide pressure arising from the up-scaling of LTNs/LLINs and IRS [13, 33, 34].

Although the overall density of *An. funestus* s.l. was higher than that of *An. gambiae* s.l, the proportion of *An. funestus* s.l. reared from field collected larvae was low compared to the proportion of *An. gambiae* s.l. This could have occurred as result of high mortality rate for the *An. funestus* larvae in the insectary during rearing due to the difficulty associated with rearing *An. funestus* [35].

For the sibling species composition of *An. gambiae* complex, our study reports *An. arabiensis* as the dominant sub-species. Previously, in the Kenyan Coast and other regions in Kenya *An. gambiae* s.s. was the dominant subspecies while *An. arabiensis* was regarded as a secondary vector [18]. However, since the up-scaling of vector control a reverse in the trends has been reported with a relative increase in *An. arabiensis* which is regarded as a more opportunistic species relative to *An. gambiae* s.s. [13, 15].

For *An. funestus* sibling species composition, this study reveals a more complex composition compared to previous studies in the area where only three subspecies were identified [36]. The present study identifies five sibling species: *An.* funestus s.s., *An. leesoni*, *An. parensis*, *An. rivulorum*, *An. vaneedeni* and six hybrids. *An. funestus* s.s. dominated the *An. funestus* population with a proportion of 76.02%. This correlates with findings from other studies in the Kenyan Coast and other regions in Kenya where *An. funestus* s.s. has been reported as the dominant *An. funestus* sibling species [36–38]. Unlike in the *An. gambiae* complex, where more exophagic and exophilic species have taken over, this study shows that *An. funestus* s.s. which is regarded as highly anthropophilic and endophagic remains the dominant sub-species. There is little information on the historical composition of *An. funestus* sub-species in the Kenyan Coast. In most of the previous studies, *An. funestus* s.l. has only been identified morphologically, thus it is difficult to tell if there has been any changes in sub-species composition over time. However, the increased complexity reported in this study compared to the two previous studies [36, 38] could be an indicator of possible changes in species composition as a result of the current vector control strategies. However, this could change with season and needs to be evaluated for different seasons and over different years before and after introduction of insecticide-treated bed nets.

In the current study, a higher proportion of malaria vectors was collected outdoor compared to indoor. This is consistent with other studies that have reported increased proportions of malaria vectors outdoor alluded to increased ITN use [39]. While change in species dominance has been linked to increased outdoor proportions, this might be the case for *An. gambiae* s.l. due to the increase in *An. arabiensis*. For *An. funestus* s.l., the dominancy of *An. funestus* s.s which is more endophagic could suggest a possible change in feeding and resting behaviours as a way to avoid insecticides. With the main vector control methods; LLINs and IRS targeting endophilic, endophagic and anthropophilic vectors, both changes in species composition and behavior adjustment pose a big threat to malaria control.

Contrary to recent studies in the area the current study reports a high overall *P. falciparum* infection rate of 4.55% [14, 19]. There are several plausible explanations for the high infection rate. First, increased insecticidal interventions over time might have led to reduced susceptibility of malaria vectors to insecticides used to treat nets. This means the nets become less effective in repelling, deterring and killing malaria vectors. Reduced efficacy of bed nets translates to increased human vector contact leading to high infection rate in mosquitoes [40]. Second, differences in sampling season could lead to differences in infection rates. Higher infection rates have been reported in drier seasons compared to wet seasons [13]. For this study mosquitoes were collected during the dry season, July to August. Third, the difference in sampling method could lead to differences in infection rates. This study used light traps and Prokopack aspirator. Light traps have been reported to increase the proportion of infected mosquitoes 2–3 times fold [41, 42]. However, the observed high infection rate might also be as a result of the changing malaria prevalence in the Kenyan Coast. Both previous studies were conducted at a time (2009–2011) when malaria prevalence was on the fall while the present study was conducted at a time when there are reports of a rising malaria prevalence in the Kenyan Coast [43].

Results from this study show that the rate of *P. falciparum* infection outdoor was the same as that of indoor. This is consistent with other studies that have reported increased *P. falciparum* infected mosquitoes outdoor with increased bed net coverage [38]. The source of outdoor sporozoite infection remains elusive since it is still not known whether the outdoor infection is as a result of the vectors resting outdoors after an infected indoor blood meal or as a result of an infected outdoor blood meal. The latter will have dire consequences on malaria control for the reason that the current vector control methods do not control outdoor biting vectors.

The 100% insecticide susceptibility exhibited by *An. funestus* yet they showed the highest level of *Plasmodium* infection seems counterintuitive. It is possible that *An. funestus* acquires the infection outdoor thus having reduced contact with insecticide treated nets. However, the possibility of insecticide resistance compromising vector competence should not be ignored as this could also be a plausible explanation for the low *P. falciparum* infection in *An. gambiae*.

## Conclusions

Taken together, results from this study report occurrence of insecticide resistance in malaria vectors in the Kenyan Coast. The presence of a low frequency of the L1014S allele in a population exhibiting phenotypic resistance calls for investigation of not only other modes of resistance but also other putative genetic markers of insecticide resistance. The occurrence of high proportions of malaria vectors outdoor highlights the need to augment ITNs and IRS with vector control methods targeting outdoor vectors.

## Abbreviations

CDC: Centre for disease control and prevention
ELISA: enzyme linked immunosorbent assay
IRS: indoor residual spraying
KEMRI: Kenya Medical Research Institute
LLINs: long-lasting insecticidal nets
NMCP: National Malaria Control Programme
PCR: polymerase chain reaction
s.l.: sensu lato
s.s.: sensu stricto
WHO: World Health Organization
